# Reduced genioglossus muscle activity caused by fluid overload in anesthetized rats

**DOI:** 10.14814/phy2.14445

**Published:** 2020-07-07

**Authors:** Parisa Sabetian, Azadeh Yadollahi, Paul B. Yoo

**Affiliations:** ^1^ Institute of Biomaterials and Biomedical Engineering University of Toronto Toronto ON Canada; ^2^ Toronto Rehabilitation Institute University Health Network University of Toronto Toronto ON Canada; ^3^ Department of Electrical and Computer Engineering University of Toronto Toronto ON Canada

**Keywords:** blood pressure, fluid overload, heart rate, obstructive sleep apnea, upper airway muscle activity

## Abstract

**Introduction:**

Although the precise cause of obstructive sleep apnea (OSA) remains unknown, various anatomical or structural factors are thought to influence upper airway patency. Recent clinical studies show that OSA is frequently observed among patients with fluid‐retaining states, such as heart/renal failure and postsurgery. It is important to note that a cause–effect relationship is not yet established, and our understanding of the effects of fluid overload is limited. The goal of this study was to investigate an animal model that can characterize the physiological changes that occur in response to fluid overload.

**Method:**

Acute nonsurvival experiments were conducted in 16 Sprague–Dawley rats. Rats were initially anesthetized by inhaled isoflurane, while the femoral vein was cannulated and urethane (1.2–1.5 g/Kg body weight) was gradually delivered intravenously to induce anesthesia. Additional doses of urethane were delivered as necessary to maintain a surgical plane of anesthesia. A surgical incision was made on the cervical area to catheterize carotid artery to measure blood pressure. A pair of stainless‐steel wires was injected into the tongue to measure genioglossus muscle activity (GGEMG). All physiological measurements were recorded as intravenous infusion of saline was provided to the rat (infusion rate = 22 ml/kg over 30 min).

**Results:**

Acute saline overloading resulted in a 33% decrease in GGEMG, when compared to baseline. There was also a gradual drop in the respiratory rate (13% decrease) that reached statistical significance at 10 min after infusion was stopped. The blood pressure exhibited a 14% increase which subsequently returned to baseline within 40 min stopping infusion. There were no significant changes in the heart rate.

**Conclusion:**

The results of this study indicate that systemic fluid overload can affect significant changes in different physiological systems including reduction in genioglossus muscle activity, increase in blood pressure, and change autonomic nervous system function.

## Graphical abstract

In this study, the effects of acute intravenous saline infusion on multiple physiological parameters—such as respiratory, cardiovascular, and autonomic nervous system—which was conducted in urethane anesthetized rodents were evaluated. Our results showed that “fluid overloading” causes a significant decrease in upper airway dilator muscle activity, which is a characteristic biomarker observed in obstructive sleep apnea patients.

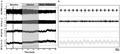

## 1 INTRODUCTION

Obstructive sleep apnea (OSA) is characterized by repeated closure—either partial or complete—of the upper airway during sleep. Obstructive sleep apnea is a common and serious disorder affecting nearly 1 billion adults aged 30–69 years worldwide (Benjafield et al., 2019). OSA is linked with excessive daytime sleepiness, impaired alertness leading to work‐related and motor vehicle accidents (George, 2001). Also, OSA contributes significantly to adverse cardiovascular events, sustained daytime hypertension, left ventricular dysfunction, and the occurrence of stroke (Arzt et al., 2005; Leung & Douglas Bradley, 2001; Peppard, Young, Palta, & Skatrud, 2000; Shepard, 1992; Yaggi et al., 2005). Although the key pathophysiological feature of OSA is repetitive collapse of the upper airway during sleep, the cause of upper airway collapse is not completely understood.

Continuous positive airway pressure (CPAP) therapy remains the gold standard therapy for treating patients with OSA. Despite the low morbidity and high efficacy of CPAP, up to 85% of patients find it difficult to tolerate (Kushida et al., 2012; Richard et al., 2007; Rosen et al., 2012). A universally known second‐line therapy does not exist; however, oral appliance therapy, upper airway reconstructive surgery, weight loss, positional therapy, and more recently, hypoglossal cranial nerve stimulation therapy can provide effective management in patients with OSA (Soose et al., 2016).

The pharyngeal muscles (e.g., genioglossus) are responsible for movement of the tongue, soft palate, uvula, and pharynx and subserve multiple functions. Essentially, pharyngeal collapse occurs when the normal reduction in pharyngeal dilator muscle tone at the onset of sleep is superimposed on a narrowed and/or highly compliant pharynx. The resulting collapse of the upper airway underscores the interaction of anatomic factors (e.g., obesity) with neural state‐related factors. Accordingly, a better understanding of the pathogenesis of OSA might lead to new and better treatments.

Although obesity is a commonly identified risk factor for OSA, it has been shown that approximately 60% of patients are in fact not obese (Soose et al., 2016). This has led to investigation of other potential factors that can contribute to upper airway obstruction. In multiple studies (Javaheri et al., 1998; Soose et al., 2016; Yadollahi et al., 2014; Young et al., 1993, 2002), OSA was found to be four times more prevalent in patients with fluid‐retaining states—such as heart and renal failure—than the general population, and this was observed despite patients having lower body weight (Arzt et al., 2006; Kimmel, Miller, & Mendelson, 1989). Also, there are studies that show early postoperative airway failure may be more closely related temporally with the fluid administration that occurs in the intraoperative period (Lam, Singh, Yadollahi, & Chung, 2016; Ramachandran, 2014). These findings raised the possibility that fluid retention may increase the risk of developing OSA. The effects of body fluid levels on upper airway function were recently tested in a double cross‐over study, where participants were given either minimum (control) or high (22 ml saline/kg body weight) levels of intravenous (IV) saline. The authors found that, when compared to control sleep sessions, in older participants, saline infusion caused significantly greater increases in neck circumference, as well as larger increases in the sleep apnea severity as assessed by the apnea–hypopnea index (AHI). There were also significant changes observed in blood pressure and heart rate variability (Vena et al., 2018). These results suggest that fluid accumulation in the neck may be significant factors that contribute to the severity of OSA symptoms.

In the current study, we used urethane anesthetized rats as an animal model for characterizing physiological changes that occur in response to fluid overload. This study involved nonsurvival experiments in which we analyzed the effects of IV saline infusion on changes in respiratory function (e.g., genioglossal muscle), arterial blood pressure, heart rate, and heart rate variability. Preliminary results were presented previously in abstract form (Yadollahi, Yoo, & Sabetian, 2017).

## 2 METHODS AND MATERIALS

### 2.1 Acute experimental set up

Experiments were conducted in 16 Sprague–Dawley rats (male, weight = 500–800 g). The protocol was approved by the Animal Care Committee at the University of Toronto and in accordance with the regulations of the Ontario Animal Research Act. Anesthesia was induced by inhaled isoflurane (5% in 100% O_2_) within an induction chamber and subsequently maintained with a gas mask (2%–3% isoflurane, O_2_ flow rate: 0.1 L/min). Following completion of all surgical procedures (described below), the anesthesia was transitioned to urethane over a period of 1 hr (initial SQ bolus: 1.2–1.5 g/kg body weight followed by supplemental doses of 0.1–0.2 g/kg.ml). Anesthetic depth was monitored throughout the experiment by measuring blood oxygen level (98%–100%), heart rate (300–350 beat/min), respiration rate, and toe pinch. The animal was euthanized at the end of each experiment by intracardiac injection of 0.3 ml/Kg T–61™ (Merck Animal Health).

### 2.2 Surgical setup

With the rat in the supine position, the carotid artery was catheterized with PE‐50 tubing (BD INTRAMEDIC™ Polyethylene Tubing, BD Medical) to measure blood pressure (BP). The catheter was connected in series with a pressure transducer (Deltran, Model: DPT‐100, Utah Med) and a syringe filled with the heparinized saline (0.5 IU/ml) that connected to an infusion pump (Model: 70‐4500, Pump11 Elite Infusion, Harvard Apparatus). The heparinized saline and infusion pumped were used to maintain a consistent pressure signal. The signal from the pressure transducer was conditioned with a bridge amplifier unit (ADInstruments) and digitally recorded (sampling rate = 10 kHz) via the data acquisition system (Powerlab 16/35, ADInstruments). The femoral vein was catheterized with PE‐50 tubing and connected in series with an infusion pump (Dual Syringe Pump, Kent Scientific) to provide intravenous (IV) infusion of saline. The electrocardiogram (ECG) was measured by subcutaneously placing needle electrodes (23G × 1″ Needles) in both forepaws and the left hind paw. A pair of unsheathed stainless‐steel wires (diameter: 0.003″ Bare, 0.0055″ coated; length: 100 Feet; A‐M systems, PFA) was implanted into the genioglossus muscle to measure the electromyogram (GGEMG). Genioglossus muscle to measure the electromyogram signals were conditioned (gain = 1000×, filter = 30 Hz to 3 kHz, MODEL SR560, Stanford Research Systems) and digitally recorded (sampling rate = 10 kHz, Powerlab 16/35, ADInstruments). A subcutaneous needle inserted in the lateral abdominal fat pad was used as the common ground.

### 2.3 Experimental protocol

All physiological parameters were measured continuously throughout each experiment. Once the anesthesia was switched to urethane, there was a 1‐hr acclimation period that was followed by the experimental protocol: (a) a 30‐min baseline (preinfusion) period, (b) a 30‐min infusion period (IV infusion of saline at 22 ml/kg), and (c) a 30‐min postinfusion period. The volume of infused saline was selected to achieve a state of fluid overload that was consistent with previous clinical experiments (Yadollahi et al., 2014), while also ensuring that infusion did not induce pulmonary edema which was confirmed by stable blood oxygen saturation levels (Schossleitner et al., 2015).

### 2.4 Data analysis

The effects of saline infusion were assessed by analyzing both directly measured (e.g., BP, ECG), and processed (e.g., heart rate (HR)) data. Analysis of the GGEMG involved Matlab (Mathworks Inc.), where the raw signal was rectified by applying a triangular Bartlett filter (window width = 300 msec) and then identified as muscle‐related activity with user‐specified adaptive threshold methods (e.g., findpeaks). The GGEMG signal was quantified by calculating the area under each detected peak. The respiratory rate was calculated by averaging the time interval between consecutive GGEMG signals within 10‐min windows (Lab chart Pro v8.1.5, ADInstruments).The HR was calculated by averaging the time interval between successive R‐peaks within 10‐min windows (Lab chart Pro v8.1.5, ADInstruments). Finally, the BP was quantified by calculating the average value within 10‐min intervals (Lab chart Pro v8.1.5, ADInstruments).

Preprocessing of the RR interval time series and computation of time and frequency domain measures of heart rate variability (HRV) were performed using Kubios HRV software (HRV 3.1.0 software (University of Kuopio) (Tarvainen, Niskanen, Lipponen, Ranta‐Aho, & Karjalainen, 2014). Artifact correction was performed to remove RR intervals that differed abnormally from the local mean RR interval. Any RR intervals greater or less than the local mean by 0.15 s were removed. The processed data were used to calculate the mean of the RR interval (mean RR), standard deviation of the RR interval (SDNN), and root mean square of successive differences (RMSSD).

Spectral analysis of HRV was performed by applying the RR intervals equidistant method (interpolation rate = 4 Hz) and a Fourier transform (Welch method) to the RR time series. The computed spectral measures were low‐frequency power (LF: 0.195–0.74 Hz), high‐frequency power (HF: 0.74–2.5 Hz), and the ratio of LF to HF (LF/HF) power (Jiang et al., 2015).

Quantitative assessment of the effects of fluid overload was based on statistical comparison of physiological variables between baseline and subsequent 10‐min intervals, defined within the infusion and postinfusion periods. A one‐way ANOVA was used to test the effect of fluid overload on individual physiological variables with a 95% confidence interval (*p* < .05). All data values presented were summarized as the mean ± *SE*.

## 3 RESULTS

**FIGURE 1:**
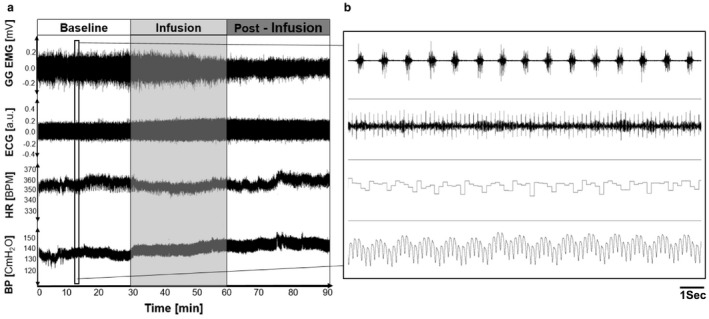
Sample data of experimentally measured variables are displayed in (a): genioglossus muscle activity (GG EMG), electrocardiogram (ECG), heart rate (HR), and blood pressure (BP). All data were segmented into (1) baseline (preinfusion, 30 min), (2) infusion (30 min), (3) postinfusion (30 min). (b) A closer examination of the data (vertical bar in a) confirms the phasic nature of GGEMG activity and the oscillatory behavior of the ECG and BP

Data were collected from 16 experiments, where genioglossus muscle activity (GGEMG), electrocardiogram (ECG), heart rate (HR), and blood pressure (BP) were measured across three time intervals (Figure 1): baseline (preinfusion, 30 min), infusion (30 min), and postinfusion (30 min).

### 3.1 Respiratory system function

**FIGURE 2:**
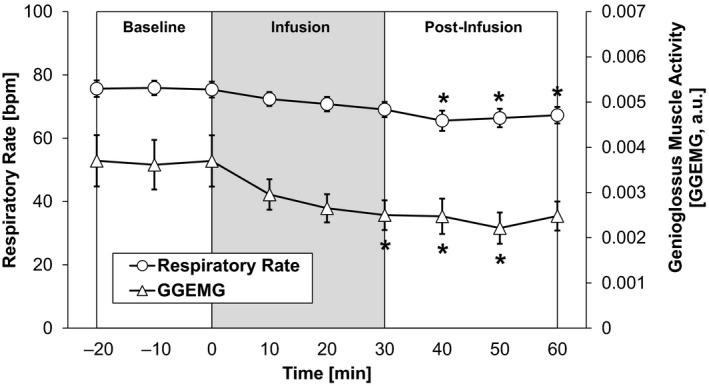
Acute IV saline infusion elicited a decrease in respiratory rate (RR) and genioglossus muscle activity (GGEMG), when compared to baseline. These changes began to occur with infusion and persisted during the postinfusion period. Each data point represents the average value of a 10‐min bin. (*n* = 16 rats), [*, significant change compared to the baseline, *p* < .05]

Acute “fluid overloading” caused significant changes in respiratory function. As shown in Figure 2, the GGEMG began to decrease with saline infusion, reaching statistical significance after 30 min of infusion (33% reduction, relative to baseline). This reduction in GGEMG was sustained for another 20 min. There was also a gradual decrease in the respiratory rate (13% decrease), which reached statistical significance 10 min after saline infusion was stopped. Both parameters returned to baseline levels within 60 min of stopping saline infusion.

### 3.2 Cardiovascular function

**FIGURE 3:**
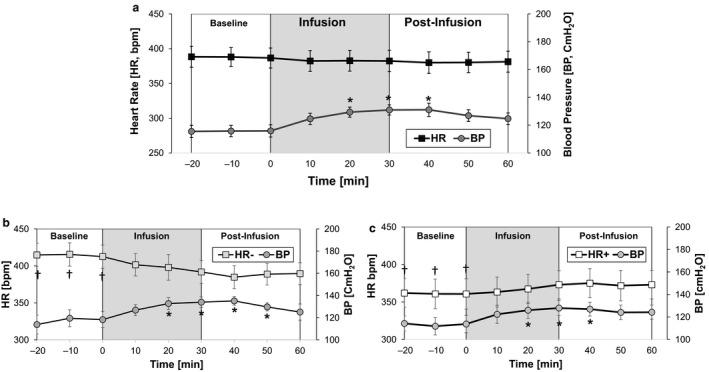
(a) Acute IV infusion of saline elicited changes in heart rate (HR) and blood pressure (BP). Based on the response to saline, animals were identified as either (b) HR^−^ (*n* = 8 rats) or (c) HR^+^ (*n* = 8 rats). In contrast, saline infusion caused an increase in BP in all 16 experiments, when compared to baseline. Each data point represents the average value of a 10‐min bin. [*, significant change compared to baseline, *p* < .05; and ^†^, significant difference between the HR^−^ and HR^+^ groups, *p* < .05)

Acute saline infusion resulted in an incremental decrease in HR (<10 bpm) during the infusion and postinfusion periods, but the changes compared to baseline not resulted in significant increase in BP, which reached statistical significance between 20 min and 40 min after infusion started (Figure 3a). In contrast, the HR exhibited a very small decrease (<10 bpm) during the infusion and postinfusion periods. Further examination of the HR data revealed two different phenotypes (Figure 3b and c): saline infusion elicited either a decrease (HR^−^ group, *n* = 8) or an increase in HR (HR^+^ group, *n* = 8). In the HR^−^ group, the initial baseline HR (414.5 ± 9.18 bpm, range: 371.5–448.7) decreased to 397.14 ± 10.53 bpm (range: 328.9–430.7) during saline infusion period and then to 387.7 ± 12.5 bpm (range: 321.8–438.8) during the subsequent postinfusion period (Figure 3b). In contrast, the HR^+^ group exhibited a baseline HR of 361.13.2 ± 19.9 bpm (range: 315–462) which increased to 367.8 ± 19.5 bpm (range: 323–469) and 373.3 ± 17.3 bpm (range: 332.5–463.2) during the infusion and postinfusion periods, respectively (Figure 3c). It is noted that there was a significant difference in the baseline HR between the HR^−^ and HR^+^ groups.

Acute saline infusion also resulted in an increase in BP across all 16 animals (Figure 3a). Compared to baseline (115.9 ± 4.37 cmH_2_O), the BP increased rapidly during infusion (128.3 ± 3.6 cmH_2_O) and remained elevated during the postinfusion period (127.5 ± 4.23 cmH_2_O). The observed increase in BP reached statistical significance (14%, compared to baseline) within 20 min of saline infusion, and remained at this elevated level for 10 min after infusion was stopped. The BP returned to baseline levels within 40 min after stopping infusion (data not plotted here).

**FIGURE 4:**
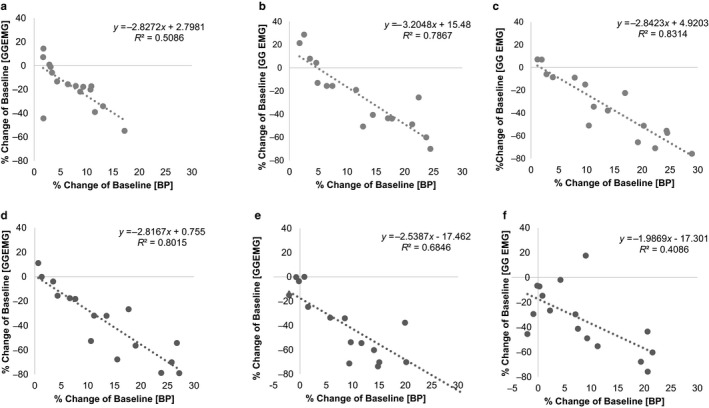
Analysis of the correlation between the percentage change in genioglossus muscle activity (GGEMG) versus the corresponding percentage change in blood pressure (BP). Both calculations were made with respect to baseline. Scatter plots are shown during saline infusion (a: first 10 min, b: second 10 min, and c: third 10 min) and the postinfusion period (d: first 10 min, e: second 10 min, and f: third 10 min). Linear regression analysis indicates a negative correlation between the calculated changes in BP and GGEMG

As shown in Figure 4, we observed a strong correlation between changes in BP and that of the GGEMG. The correlation between the two biomarkers reached maximum values during the final 10 min of saline infusion period (Figure 4c, *R* ^2^ = .83) and the initial 10 min of the postinfusion period (Figure 4d, *R* ^2^ = .80). By the end of the postinfusion period (Figure 4f), the correlation returned to baseline condition (Figure 4a).

### 3.3 Autonomic function

**FIGURE 5:**
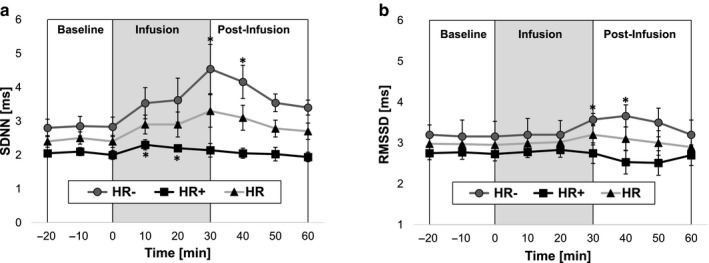
Time‐domain analysis of heart rate variability (HRV). Data were plotted for the three different phases (baseline, infusion, and postinfusion) as average values of 10‐min bins. (a) Significant changes in SDNN were observed in both groups, where the HR^−^ group showed a peak 60% increase at 30 min and the HR^+^ group showed a 15% increase at 10 min after initiating saline infusion. (b) A significant increase in RMSSD (10%, compared to baseline) was observed only in the HR^−^ group, where statistical significance was reached between 30 and 40 min after saline infusion was initiated. [*, represents the significant changes compared to the baseline, *p* < .05]. HR^+^ (*n* = 8 rats) and HR^−^ (*n* = 8 rats)

Although time‐domain analysis of HRV using all experimental data (*n* = 16) revealed that saline infusion did not elicit any significant changes, we found there were significant changes in autonomic activity that occurred in the HR^−^ and HR + groups. As shown in Figure 5a, analysis of the SDNN indicated significant increases in sympathetic activity in both the HR^−^ group (time = 30–40 min) and the HR^+^ groups (time = 10–20 min). In both groups, the SDNN returned to baseline levels within 20 min of stopping infusion. Analysis of the RMSSD (Figure 5b) indicated that parasympathetic activity increased only in the HR^−^ group (Time = 30 to 40 min), which subsequently returned to baseline by the end of the postinfusion period. Interestingly, this finding suggests that there was an approximately 30‐min delay between saline infusion and vagal‐mediated changes in cardiac function. Analysis of the mean RR interval showed there were no significant changes in either the HR^−^ or HR^+^ groups (data not shown).

**FIGURE 6:**
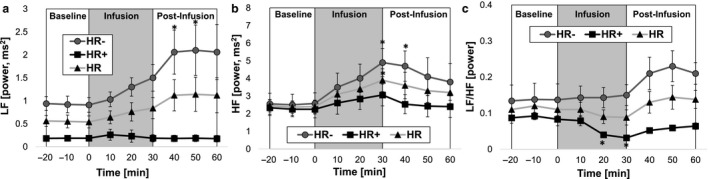
Frequency‐domain analysis of heart rate variability (HRV): (a) low‐frequency (LF) power, (b) high‐frequency (HF) power, and (c) the ratio of the low‐frequency to high‐ frequency (LF/HF) power. Data were plotted for the three different phases (baseline, infusion, and postinfusion) as average values of 10‐min bins. Significant increases in LF and HF power were observed in HR^−^ group (a and b); whereas, the HR^+^ group exhibited significant changes only with respect to LF/HF ratio (c). [*, represents the significant changes compared to the baseline, *p* < .05]. HR^+^ (*n* = 8 rats) and HR^−^ (*n* = 8 rats)

Frequency domain analysis yielded similar results to that of our time‐domain analysis. As depicted in Figure 6a and b, the HR^−^ group exhibited significant increases in LF power at t = 40 min (126% increase), along with significant increases in HF power at t = 30 min (102% increase). The change in the LF/HF ratio was not significant (Figure 6c). In contrast, there were no significant changes in LF and HF power in the HR^+^ group (Figure 6a and b), but the significant decrease in the LF/HF ratio suggested a parasympathetic dominance during the infusion period (Figure 6c).

## 4 DISCUSSION

In this study, we investigated the effects of acute IV saline infusion on multiple physiological parameters—such as respiratory, cardiovascular, and autonomic nervous activity—in urethane anesthetized rodents. Our results showed that “fluid overloading” (infusion rate = 22 ml/kg, 30 min) caused a significant decrease in upper airway dilator muscle activity, which is a characteristic biomarker observed in OSA patients (Remmers, deGroot, Sauerland, & Anch, 1978). This change occurred during infusion and persisted for approximately 30 min, before eventually returning to baseline levels. In conjunction with the GGEMG, there was a corresponding increase in BP that occurred during and after saline infusion. The hypertensive effects of infusion observed in this study were consistent with previous work in dogs (Coleman & Guyton, 1969; Vatner, Boettcher, Heyndrickx, & McRitchie, 1975), rabbits (Maybaum, Gorodetsky, & Weinstock, 1998), and humans (Fujimoto et al., 2013; Vena et al., 2018; Yadollahi et al., 2014).

This is the first study to examine quantitatively the relationship between BP and GGEMG in an anesthetized rat model. As depicted in Figure 4, there was a clear negative correlation between the two variables which became strongest (peak *R* ^2^ value) as the BP reached maximum values. Our findings are consistent with previous work which show that increases in blood pressure result in reduced diaphragm activity and total ventilation (Metcalfe, Chew, Clarke, de Donaldson, & Taylor, 2015; Salamone, Strohl, Weiner, Mitra, & Cherniack, 1983; Tan et al., 2014), though it remains unclear whether saline infusion can affect GGEMG independent of changes in BP. Given that GGEMG activity parallels ventilation in general, the significant decrease in respiratory rate during the postinfusion period (Figure 2) was expected. It is noted that our analysis of BP versus GGEMG is also consistent with animal and clinical studies involving pharmacologically induced states of hypertension (Garpestad et al., 1992; Trelease, Sieck, Marks, & Harper, 1985). The negative correlation between BP and GGEMG shown here can also have important implications for describing the mechanism of recurrent apnea‐related events—such as hypoxia and arousal—which lead to acute rises in arterial blood pressure (Field, White, & Lang, 1993).

Another interesting outcome of this study was the identification of HR^+^ and HR^−^ rats, which was based on the observed change in HR caused by saline infusion. The respective HR of each group at baseline was statistically different, but later converged following infusion (Figure 3b and c). Our analysis of HRV further showed how the autonomic nervous system of each group responded very differently to saline infusion. The HR^−^ group exhibited clear signs of elevated activity of vagal tone (RMSSD and LF component) along with signs of elevated sympathetic activity (LF/HF ratio) following saline infusion (Shaffer & Ginsberg, 2017). The HR^+^ group exhibited signs on elevated sympathetic activity (SDNN) which was consistent with the observed increase in HR.

Possible factors that may account for the differential changes in HR may include variability in the sensitivity to and metabolism of the injected urethane anesthesia among animals. Previous studies show that urethane anesthetized rats exhibit a HR in the range of 320–420 bpm, but dose and route of administration can have significant effects (Carruba, Bondiolotti, Picotti, Catteruccia, & Da Prada, 1987; Field et al., 1993; Maggi & Meli, 1986). For example, urethane doses of 1.2 g/kg and 1.5 g/kg in rats can result in 15% and 20% reductions in HR, respectively, when compared to baseline. Although not examined specifically in this study, the initial use of inhaled isoflurane (e.g., duration of use during surgical procedures) could have also interacted with subsequent administration of urethane. Another potential factor involves the surgical manipulation of the vagus nerve during carotid artery catheterization, since it has been shown that vagal stimulation can induce bradycardia in urethane anesthetized rats (Hotta et al., 2009).

This current study showed that acute “fluid overloading” causes a consistent change in both BP and GGEMG which were in turn shown to be strongly correlated in a negative manner. As indicated by our HRV analysis, these physiological responses can be linked to changes in the autonomic nervous system. Previous work in human subjects report that intravenous fluid overload causes a small (ΔHR = −5.2 ± 9.2 bpm) but nonsignificant decrease in HR (Vena et al., 2018; Yadollahi et al., 2014). Although the human participants were not grouped into HR^+^/HR^−^ cohorts, the standard deviation of the ΔHR suggests there was considerable variability among participants. Therefore, when considering the mean decrease in HR observed in humans, the HR^−^ group provided the closest approximation in terms of physiological responses elicited by saline infusion. In addition to diminished GGEMG activity (linked to increased susceptibility to OSA in humans), rats in the HR^−^ group exhibited similar cardiac autonomic regulation in the form of temporal and spectral measures of HRV. This study suggests that our HR^−^ model could potentially be used to investigate the role of HRV in assessing risk stratification for cardiovascular events and mortality in sleep apnea patients. By incorporating upper airway obstructions into our animal model, future work may be able to predict the decreased cardiac vagal modulation that is observed in OSA patients during sleep (Horner et al., 1996; Khoo, Kim, & Berry, 1999; Penzel, Kantelhardt, Grote, Peter, & Bunde, 2003). Reduced HRV and vagal withdrawal are associated with an increased risk for cardiovascular events in a general population (Tsuji et al., 1996) and increased mortality in patients with post myocardial infarction (Bigger, Fleiss, Rolnitzky, & Steinman, 1993; Kleiger, Miller, Bigger, & Moss, 1987). Further work is also needed to examine the physiological effects of fluid overload in more sophisticated animal models of OSA.

## 5 CONCLUSIONS

Our findings indicate that acute episodes of fluid overload can significantly reduce genioglossus muscle activity in anesthetized rats and therefore should be considered a potentially useful animal model for investigating the pathophysiology of OSA. The novel animal model presented here could be further validated by measuring fluid accumulation in peripharyngeal tissue, assessing changes in upper airway dilator reflexes, and by also exploring these phenomena in larger animal models. Testing this model under different experimental conditions—use of alternative anesthetic agents (e.g., α‐chloralose) or fully awake animals—may provide significant insight into the pathological influence of fluid overload in OSA.

## CONFLICT OF INTEREST

The authors declare no conflict of interest.

## AUTHOR CONTRIBUTIONS

P. Sabetian, A. Yadollahi, and P. Yoo: designed and performed experiments, analyzed data, and co‐wrote the paper. P. Sabetian, P. Yoo: performed the experiments and data analysis, drafted the manuscript, and designed figures. A. Yadollahi, P. Yoo: provided essential guidance and aided in interpreting the results. A. Yadollahi, P. Yoo: supervised the research. All authors discussed the results and commented on the manuscript and co‐wrote the paper.
